# Cell release during perfusion reflects cold ischemic injury in rat livers

**DOI:** 10.1038/s41598-020-57589-4

**Published:** 2020-01-24

**Authors:** Reinier J. de Vries, Casie A. Pendexter, Stephanie E. J. Cronin, Beatriz Marques, Ehab O. A. Hafiz, Alona Muzikansky, Thomas M. van Gulik, James F. Markmann, Shannon L. Stott, Heidi Yeh, Mehmet Toner, Korkut Uygun, Shannon N. Tessier

**Affiliations:** 1 Center for Engineering in Medicine, Harvard Medical School and Massachusetts General Hospital, Boston, MA USA; 2 Shriners Hospitals for Children - Boston, Boston, MA USA; 3 Department of Surgery, Amsterdam University Medical Centers – location AMC, Amsterdam, the Netherlands; 4 Department of Electron Microscopy Research, Theodor Bilharz Research Institute, Giza, Egypt; 5 Biostatistics Center, Massachusetts General Hospital, Boston, MA USA; 6 Cancer Center, Massachusetts General Hospital and Harvard Medical School, Boston, MA USA; 7 Division of Transplant Surgery, Massachusetts General Hospital, Boston, MA USA

**Keywords:** Mechanisms of disease, Predictive markers, Liver, Translational research

## Abstract

The global shortage of donor organs has made it crucial to deeply understand and better predict donor liver viability. However, biomarkers that effectively assess viability of marginal grafts for organ transplantation are currently lacking. Here, we showed that hepatocytes, sinusoidal endothelial, stellate, and liver-specific immune cells were released into perfusates from Lewis rat livers as a result of cold ischemia and machine perfusion. Perfusate comparison analysis of fresh livers and cold ischemic livers showed that the released cell profiles were significantly altered by the duration of cold ischemia. Our findings show for the first time that parenchymal cells are released from organs under non-proliferative pathological conditions, correlating with the degree of ischemic injury. Thus, perfusate cell profiles could serve as potential biomarkers of graft viability and indicators of specific injury mechanisms during organ handling and transplantation. Further, parenchymal cell release may have applications in other pathological conditions beyond organ transplantation.

## Introduction

End-stage liver disease contributes to 77,000 deaths annually in the US alone^1^ and transplantation is often the only treatment option. Due to the severe donor organ shortage, merely 12,000 patients are listed on the liver transplant wait-list and of these, only 8,000 will receive a transplant each year in the US^2^. Thus, improving access to this lifesaving treatment has become an immediate necessity. Paradoxically, the donor organ shortage is not caused by a limited availability of cadaveric organs — more than 25% of donor livers procured for transplantation are not ultimately transplanted^3^. Also, it is estimated that there is an additional donor pool of 6,000 unprocured livers/year, of which many are only marginally damaged^4^. However, because of their uncertain viability, none of these potential donor organs are used while transplantation of just a fraction of these organs could dramatically reduce the organ shortage.

The current lack of quantitative, objective, and graft-specific diagnostics to effectively evaluate graft viability and accurately predict transplant outcome has critically limited the availability of livers for transplantation. Instead, current clinical standards use only gross population-based donor risk statistics (such as age, race, height, among others)^5,6^. Because of such non-specific indicators coupled with the unacceptable costs of an unsuccessful transplant, perfectly good donor livers are discarded every year. Identifying novel biomarkers that can predict safe utilization of the grafts that are currently discarded would significantly help relieve the donor organ shortage.

Latest advances in machine perfusion have offered several solutions to overcome the organ shortage, including *ex vivo* viability assessment of marginal donor organs. However, clinical accurate assessment of liver viability during machine perfusion is elusive and would benefit from additional viability biomarkers^7–9^. Further, fundamentally different *ex vivo* machine perfusion modalities aimed at resuscitating marginal organs have emerged with unique pros and cons^10,11^ and understanding of the specific injury mechanisms of each cell type may help improve the different machine perfusion and preservation technologies.

Liver-specific cell types can be categorized as structural or resident immune cells, and both could be promising candidates for assessing organ injury. Structural liver cells such as liver sinusoidal endothelial cells (LSECs), hepatocytes, and liver stellate cells typically stay in the liver under normal physiological conditions. However, upon liver injury, we hypothesize that they are likely to get released due to their anatomical location near the sinusoidal capillaries^12^. Further, the liver is home to three types of resident immune cells that were assessed in this study^13,14^: (1) Kupffer cells, (2) liver-specific natural killer cells (also known as pit cells^15^), and (3) dendritic cells. Because tissue injury is either primarily caused by or secondarily evokes an immune reaction^16–18^, detectable alterations in the immune cells that are released from the organ may correlate with tissue injury and organ viability.

We propose that these organ-specific cells are released during graft handling and preservation and may be novel candidates for assessing the fitness of an organ prior to transplantation. To our knowledge, whole cell release from organs in response to injury and its implications on graft viability have not been studied before. Organ-specific cells could be promising biomarkers because: (1) they can be sampled non-invasively; (2) unlike tissue biopsies, these cells represent the whole organ and capture spatial differences in tissue injury; (3) they can be easily obtained from the flush after hypothermic preservation or from the perfusate during machine perfusion; (4) unlike other blood-based biologics such as cell-free DNA, microparticles, and/or exosomes, these cells are not abundantly shed from normal tissues and can thus be used to specifically identify injury-derived expression signatures; (5) the functional specificity of each cell type could be leveraged to identify and understand complex injury mechanisms.

In summary, the objective of this study is to investigate the release of liver specific cells as a result of ischemic injury during hypothermic preservation (+4 °C). Here, we present a method for the isolation and characterization of rat liver-derived cells from perfusates. We show that both structural and resident immune cells are released from injured livers and that their release significantly changes as function of cold ischemia (CI) duration.

## Results

### Total cell release during machine perfusion as a function of cold ischemia duration

**Figure 1:**
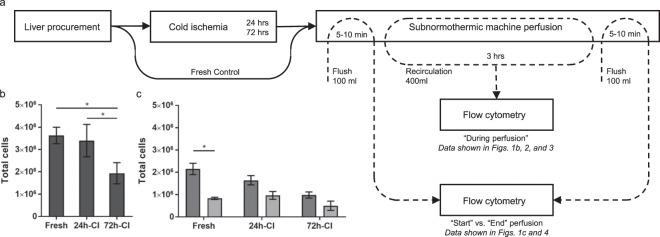
Experimental design and total number of released cells in the perfusates. (**a**) Schematic representation of the research design. The perfusates of fresh (n = 4), 24-h-cold ischemic (CI) (n = 5) and 72-h-CI (n = 4) rat livers that recirculated for 3 h *during* subnormothermic machine perfusion was collected and analyzed using multi-channel imaging flow cytometry. Fresh fractions of the perfusate that flushed through the liver at the *start* and *end* of perfusion were collected and analyzed separately. The experimental CI groups correspond to the previously established CI limit for 100% transplant survival (24-h-CI) and 0% transplant survival (72-h-CI) in rats after SNMP^20,21^. (**b**) Total number of cells released *during* perfusion. (**c**) Total number of cells released in the flushes at the *start* (dark gray) and *end* (light gray) of perfusion. Error bars: SEM. Stars denote statistical significance (repeated measures two-way ANOVA, followed by Tukey’s post-hoc test): *0.01 < p < 0.05.

**Figure 2:**
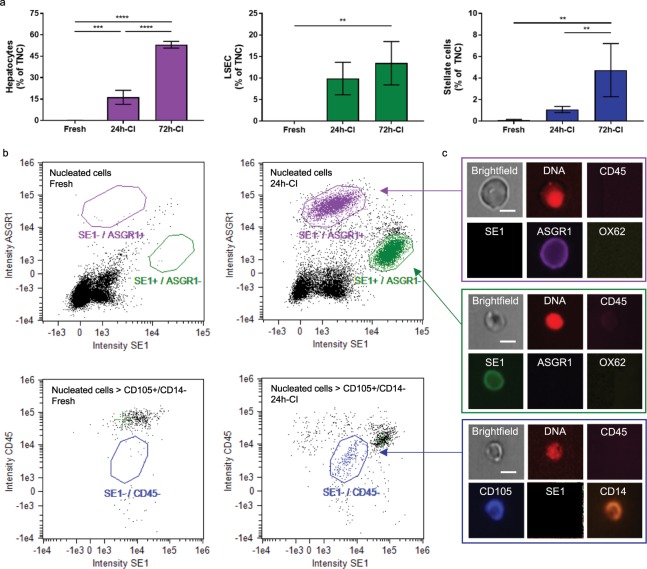
Release of structural liver cells into the perfusate after cold ischemia. (**a**) Percentages of *presumed* hepatocytes (left/purple), sinusoidal endothelial cells (middle/green), and stellate cells (right/blue) in the perfusate, relative to the total number of nucleated cells (TNCs) in the perfusate. The perfusate recirculated during 3 hours of subnormothermic machine perfusion. Stars denote statistical significance (two-way ANOVA, followed by Tukey’s post-hoc test): *0.01 < p < 0.05; **0.001 < p < 0.01; ***0.0001 < p < 0.001; ****p < 0.0001. Error bars: SEM. (**b**) Imaging flow cytometry for quantification of *presumed* hepatocytes (CD45−/SE1−/ASGR1+/OX62−), liver sinusoidal endothelial cells (LSECs) (CD45−/SE1+/ASGR1−/OX62−), and stellate cells (CD45−/CD105+/SE1−/CD14+). Left: fresh livers. Right: 24-h-CI livers. (**c**) Representative images of surface marker expression of hepatocytes (top), LSEC (middle), and stellate cells (below). Scale bars: 5 µm.

**Figure 3:**
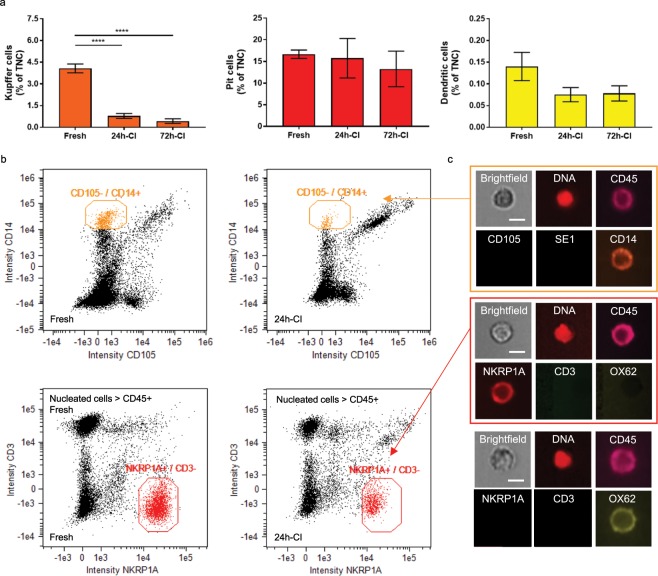
Alterations in liver-resident immune cell release into the perfusate after cold ischemia. (**a**) Percentage of *presumed* Kupffer cells (left/orange), liver-resident natural killer cells (also known as pit cells) (middle/red), and dendritic cells (right/yellow) in the perfusate, relative to the total number of nucleated cells (TNCs) that are released into the perfusate from fresh (n = 4), 24-h-cold ischemic (CI) (n = 5), and 72-h-CI (n = 4) livers. The perfusate recirculated during 3 hours of subnormothermic machine perfusion. Stars denote statistical significance (two-way ANOVA, followed by Tukey’s post-hoc test): *0.01 < p < 0.05; **0.001 < p < 0.01; ***0.0001 < p < 0.001; ****p < 0.0001. Error bars: SEM. (**b**) Imaging flow cytometry for the quantification of *presumed* Kupffer cells (CD45+/CD105−/SE1−/CD14+) and pit cells (CD45+/NKRP1A+/CD3−/OX62−). Dendritic cells were manually selected from a CD45+/NKRP1A−/CD3−/OX62+ population (not shown). Left: fresh livers. Right: 24-h-CI livers. (**c**) Representative images of surface marker expression images of Kupffer cells (top), pit cells (middle), and dendritic cells (below). Scale bars: 5 µm.

**Figure 4:**
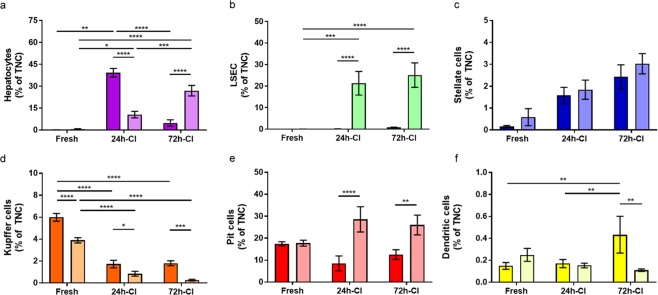
Differences in cell release between perfusates collected at the start and end of perfusion. Percentages of liver specific cells released into fresh fractions of perfusate that flushed once through the liver at the start (dark colors) and end (bright colors) of perfusion. These fractions were collected separately from the perfusate that recirculated during 3 hours of subnormothermic machine perfusion and were analyzed to study the change in cell release. (**a**) *Presumed* hepatocytes (purple). (**b**) *Presumed* sinusoidal endothelial cells (green). (**c)** *Presumed* stellate cells (blue). (**d**) P*resumed* Kupffer cells (orange). (**e**) *Presumed* pit cells (red). (**f**) *Presumed* dendritic cells (yellow). Percentages are relative to the total number of nucleated cells (TNCs). Stars denote statistical significance (repeated measures two-way ANOVA, followed by Tukey’s post-hoc test): *0.01 < p < 0.05; **0.001 < p < 0.01; ***0.0001 < p < 0.001; ****p < 0.0001). Error bars: SEM.

The clinical standard for organ preservation is hypothermic preservation (HP) at 4 °C in a specialized preservation solution such as the University of Wisconsin solution (UW)^19^. For rat livers, the maximum viable HP duration is 24 h^20^. We have previously shown that extending the duration of CI leads to a sharp decline in organ viability, resulting in 0% transplant survival after 72 h of HP, despite a 3-h subnormothermic machine perfusion (SNMP) resuscitation^20,21^. Therefore, we chose to study cell release from rat lives after these two CI durations to represent transplantable (24-h-CI) vs. non-transplantable (72-h-CI) rat livers, in addition to a fresh control. Following CI, all livers were subjected to 3-h SNMP. We isolated cells from the perfusate that recirculated during SNMP and analyzed them using imaging flow cytometry. We refer to this as cell release *“during perfusion”* and the corresponding data is shown in Figs. 1b, 2 and 3. Additionally we flushed the livers with separate fresh perfusate fractions at the *“start of perfusion”* and *“end of perfusion”*, to study the cell release in the flush directly after HP and the difference in cell release over time. The corresponding data is shown in Figs. 1c and 4. The research design is schematically shown in Fig. 1 and explained in detail in the Materials and Methods.

We first studied if and how many cells were released from fresh and injured liver grafts by analyzing the total number of cells released from fresh, 24-h-CI and 72-h-CI livers during perfusion. We found that over 3 million cells were released from fresh (n = 4) and 24-h-CI (n = 5) livers during perfusion (3.64 ± 0.74 million (M) and 3.40 ± 1.63 M, respectively; mean ± standard deviation (SD) throughout the text, unless specified), providing ample cell quantities for downstream analysis (Fig. 1b). Cell release from 72-h-CI (n = 4) livers was significantly lower (1.94 ± 0.95 M; repeated measures two-way ANOVA followed by the Tukey post-hoc test throughout the text unless otherwise specified) than fresh and 24-h-CI livers (p = 0.0114 and p = 0.0225, respectively). Further, we found that white blood cells (i.e. CD45+ cells) accounted for a considerable percentage of cells that declined in number as a function of CI time (94.6 ± 5.92%, 51.2 ± 19.65%, 15.8 ± 6.27% in fresh, 24-h-CI, 48-h-CI, and 72-h-CI livers, respectively; Table S1). We suggest that a significant portion of this white blood cell population originates from peripheral blood rather than from the liver because only ~13–21% of the released cells were identified as liver-specific immune cells.

### Release of structural liver cells during machine perfusion

After confirming that millions of cells are released during perfusion, we used imaging flow cytometry to characterize the cell types released from the liver grafts during perfusion. Cell type numbers were expressed and analyzed as a percentage of the total number of nucleated cells (TNCs) released in the perfusate (the absolute values are presented in Table S2). For analyzing structural liver cells, we used ASGR1 as a marker for hepatocytes^22–24^; however, it should be noted that 5–10% of ASGR1− positive cells have been reported as fibroblasts in human liver biopsies^24^. For LSECs, we used the rat-specific sinusoidal endothelial cell marker SE1^25,26^. Stellate cells express CD105^27^; however, this surface marker can also be expressed on LSECs^28,29^. Therefore, CD105-positive and SE1-negative cells were selected as stellate cells (Fig. 2c). ASGR1+, SE1+, and CD105+/SE1− cells are referred to as *presumed* (denoted by p in superscript) hepatocytes^p^, LSECs^p^, and stellate^p^ cells, respectively in the text.

All three types of structural liver cells were nearly absent in the perfusates of fresh livers. After 24 h and 72 h of CI, increasing numbers of all three types of structural liver cells were released into the perfusate (Fig. 2a). Intensity plots of the specific surface markers (Fig. 2b) clearly showed distinct hepatocyte^p^, LSEC^p^, and stellate^p^ cell populations in the CI groups, but not in the fresh controls.

This increased release of structural liver cells after CI was most evident for hepatocytes^p^, which showed significantly different percentages in the perfusates of fresh (0.12 ± 0.05%), 24-h-CI (16.32 ± 11.01%) and 72-h-CI (53.06 ± 4.70%) livers (p < 0.0001 between all groups). LSEC^p^ release showed a similar trend, with a significantly higher percentage of LSECs^p^ in the perfusates of 72-h-CI livers (13.46 ± 10.05%) compared with that of fresh (0.01 ± 0.00%) livers (p = 0.0350). The percentages of released stellate^p^ cells (Fig. 2a) were much lower (0.11 ± 0.11%, 1.08 ± 0.64% and 4.74 ± 4.94% for fresh, 24-h-CI, and 72-h-CI livers, respectively) than those of hepatocytes^p^ and LSECs^p^. However, a significant increase in the percentage of stellate^p^ cells was found in 72-h-CI livers compared with fresh and 24-h-CI livers (p = 0.0019 and p = 0.0099, respectively). Together, these results confirm that tissue injury leads to the release of structural liver cells. More importantly, it shows that the release of structural liver cells is significantly different between livers with no, mild, and severe ischemic injury.

### Release of liver-resident immune cells during machine perfusion

To analyze the percentages of liver-resident immune cells in the perfusate, the combined expression of the general white blood cell marker CD45 with specific membrane markers was used to identify each of the three immune cell types (Fig. 3c). In addition to using CD45, we identified Kupffer cell by CD14 surface antigen expression^30–32^, Pit cells by expression of the general natural killer cell marker NKRP1A and the absence of CD3^33,34^, and dendritic cell by the expression of OX62 and the absence of CD3^35^. In this context, the *presumed* Kupffer^p^, pit^p^, and dendritic^p^ cells are referred to as CD45+/CD14+, CD45+/NKRP1A+/CD3−, and CD45+/OX62+/CD3− cells, respectively.

In contrast to the trend observed in structural liver cells, the percentage of Kupffer^p^ cells in the perfusate of fresh livers was high, but decreased with increasing durations of CI (Fig. 3a). We observed a sharp decline in Kupffer^p^ cell release in the 24-h-CI (0.78 ± 0.39%) and 72-h-CI (0.42 ± 0.33%) livers compared with fresh livers (4.07 ± 0.60%; p < 0.0001 and p < 0.0001, respectively). Both the percentage (Fig. 3a) and absolute number (Table S2) of Kupffer^p^ cells in the 24-h-CI and 72-h-CI groups decreased by approximately two- and three-fold, respectively, compared with the fresh liver group; however, this result did not reach statistical significance.

Pit^p^ cells accounted for the largest population of released liver-resident immune cells and showed similar percentages for all experimental groups (16.66 ± 1.95%, 15.74 ± 10.19%, and 13.28 ± 8.22% for fresh, 24-h-CI, and 72-h-CI livers, respectively). Dendritic^p^ cells are a rare cell type in the liver, which was consistent with the low percentages of dendritic^p^ cells (Fig. 3a**)** that were released into the perfusate (0.14 ± 0.07%, 0.08 ± 0.04%, and 0.08 ± 0.04 for fresh, 24-h-CI, and 72-h-CI livers, respectively). No statistical differences were found between the liver groups for dendritic^p^ cells.

Significant alterations in the liver-resident immune cell profiles during perfusion were observed following CI. Although these alterations did not significantly differentiate between moderately and severely injured CI livers, they could differentiate between fresh and CI livers.

### Differences between the release of structural liver cells at the start and end of perfusion

After analysis of the cells that were released in the perfusate that recirculated during perfusion, we now present and compare the release of liver specific cells derived from separate perfusion fractions that were flushed through the livers at the start of perfusion and at the very end of perfusion, as shown in the research design in Fig. 1.

No hepatocytes^p^ were released at the start of perfusion in fresh livers (0.11 ± 0.06%), whereas after 24 h of CI, a significantly higher percentage of the released nucleated cells were hepatocytes^p^ (39.19 ± 6.65%; p < 0.0001) (Fig. 4a). Interestingly, the percentage of hepatocytes^p^ at the start of perfusion in 72-h-CI livers (4.79 ± 4.36%) was significantly lower than that in 24-h-CI livers (p < 0.0001). Additionally, the percentage of hepatocytes^p^ was higher at the start than at the end of perfusion in the 24-h-CI group (39.19 ± 6.65% vs. 10.54 ± 5.08%; p < 0.0001), and the opposite trend was observed in the 72-h-CI group (4.79 ± 4.36% vs. 26.93 ± 7.24%; p < 0.0001) (Fig. 4a). This may be caused by the lysis of injured hepatocytes after 72 h of CI or may also indicate delayed onset of hepatocyte detachment during perfusion as a function of ischemic injury.

Almost no LSECs^p^ were released at the beginning of perfusion in both the fresh controls and the 24-h- and 72-h-CI groups (0.02 ± 0.02%, 0.12 ± 0.10%, and 0.80 ± 0.30%, respectively) (Fig. 3b). The LSEC^p^ percentages at the beginning of perfusion were very low and were not significantly different between the three liver groups. Almost no LSECs^p^ were released at the end of perfusion in fresh livers (0.05 ± 0.07%). Interestingly, LSEC^p^ percentages in both the 24-h- and 72-h-CI livers were significantly higher at the end than at the start of perfusion (21.32 ± 12.29%; p = 0.0004 and 25.11 ± 11.32%; p < 0.0001, respectively).

The percentages of stellate^p^ cells in the perfusate were low at the start (0.15 ± 0.10%, 1.58 ± 0.83% and 2.43 ± 1.10% for fresh, 24-h-CI, and 72-h-CI livers, respectively) and slightly higher at the end (0.56 ± 0.78%, 1.84 ± 0.99 and 3.03 ± 0.92% for fresh, 24-h-CI, and 72-h-CI livers, respectively) of perfusion (Fig. 4c). However, the differences between the groups did not reach statistical significance.

### Release of liver-resident immune cells at the start and end of perfusion

We hypothesized that the release of liver-resident immune cells, unlike that of structural liver cells, would be high at the start and lower at the end of perfusion due to a ‘wash-out’ effect during perfusion. This was confirmed by the significantly higher percentage of Kupffer^p^ cells at the beginning than at the end of perfusion in fresh, 24-h-CI, and 72-h-CI liver perfusates (5.99 ± 0.70% vs. 3.89 ± 0.48%, p < 0.0001; 1.74 ± 0.77% vs. 0.85 ± 0.52%, p = 0.0300; and 1.80 ± 0.45% vs. 0.26 ± 0.16%, p = 0.0009, respectively) (Fig. 3d). Kupffer^p^ cell percentages in the perfusates flushed at the start of perfusion significantly decreased with increasing duration of ischemia (p < 0.0001 for both fresh vs. 24-h-CI and fresh vs. 72-h-CI livers), which was similar to the perfusate that was collected during perfusion (Fig. 3a). The difference between the release of pit^p^ cells at the start and end of perfusion from cold ischemic livers showed an opposite trend to that of Kupffer^p^ cells (Fig. 4d). For both the 24-h-CI and 72-h-CI grafts, the percentage of pit^p^ cells was significantly lower at the start than at the end of perfusion (8.53 ± 7.60% vs. 28.60 ± 12.85%, p < 0.0001 and 12.58 ± 4.42% vs. 26.03 ± 9.02%, p = 0.0027, respectively) (Fig. 4e).

The percentage of dendritic^p^ cells released at the start of perfusion was significantly higher for the 72-h-CI group (0.43 ± 0.34%) compared with that of both the fresh (0.14 ± 0.06%) and the 24-h-CI (0.17 ± 0.08%) groups (p = 0.0072 and p = 0.0089, respectively). The percentage of dendritic^p^ cell release at the end of perfusion was the same regardless ischemic injury (Fig. 4f).

### Liver function and injury during machine perfusion as result of cold ischemia

One important benefit of machine perfusion is that it allows the *ex vivo* assessment of liver viability. Although some of these parameters have been clinically correlated to transplant outcomes after normothermic machine perfusion, this remains to be determined for SNMP^7,8,20,36,37^. Nonetheless, the different biochemical and mechanical parameters have strong theoretical background and are informative of liver function and injury during SNMP.

Liver weight is an indirect measure of cellular edema and we did not find significant differences in liver weights between the three groups (Fig. S1a). Microcirculatory dysfunction due to endothelial and stellate cell injuries, can cause increased vascular resistance^38^. The vascular resistance (Fig. S1b) of the 72-h-CI livers was significantly higher than both the fresh (p < 0.0339 at all time points) and the 24-h-CI (p < 0.0385 at t = 0; 120; 150; 180 min) livers. Likewise, fresh livers had significantly lower perfusion pressures (Fig. S1c) than the 24-h-CI livers (p < 0.0015 at all time point after t = 0 min) and 72-h-CI livers (p < 0.0001 at all time points), while the flow (Fig. S1d) was significantly lower in the 72-h-CI livers as comparted to the fresh (p < 0.0347 at all time points after t = 30 min) and the 24-h-CI livers (p < 0.0152 at all time points after t = 90 min). Together this is indicative of significant endothelial injury in the 72-h-CI livers, which was consistent with our previous findings which correlate vascular resistance and transplant survival of rat livers after SNMP^20,21^.

Both bile production and oxygen uptake are (hepato)cellular metabolic activity parameters which – despite the reduced metabolic rate at 21 °C – are normally observed during SNMP^20,21,39–41^. The cumulative bile production (Fig. S1e) was at the highest levels in fresh (41.63 ± 5.88 µl g^−1^), at intermediate levels in the 24-h-CI livers (18.26 ± 7.99), and was nearly absent in the 72-h-CI (0.62 ± 0.13) livers (p < 0.0001 at the end of perfusion between all groups). The oxygen uptake (Fig. S1f) followed the same trend, albeit significant differences in total oxygen uptake (area under the curve, AUC) was only observed between the fresh (3.55 ± 0.70 l/g) and 72-h-CI livers (1.87 ± 0.20; p = 0.0061, Kruskal-Wallis test on AUC followed by the Dunn’s post-hoc test).

Besides liver function and metabolic metrics such as bile production and oxygen uptake, we also assessed important parameters of liver injury in the venous outflow during perfusion^7^. Whereas release of AST indicates hepatocyte-specific injury (Fig. S1g), potassium is a general parameter of cellular injury (Fig. S1h). Both the AST and potassium levels were highest in the perfusate of the 72-h-CI livers. The levels were the highest at the very start of perfusion (t = 0 min), and directly dropped at t = 30 min, due to a feature of the experimental design whereby the liver is flushed with 100 ml of perfusate to collect cells for imaging flow cytometry (i.e. the “*start of perfusion*” fraction, see schematic design in Fig. 1a). Whereas the AST activity of fresh and 24-h-CI livers remained similar and stable during perfusion, the AST activity of the 72-h-CI livers increased over time, indicative of ongoing hepatocellular injury during perfusion, which resulted in significantly higher values (1.38 ± 0.59 U l^−1^ g^−1^) compared with the fresh (0.35 ± 0.17) and the 24-h-CI (0.28 ± 0.08) livers at the end of perfusion (p = 0.0109 and p = 0.0036, respectively at t = 180 min). The potassium levels also peaked at the start of perfusion in the 72-h-CI livers (8.43 ± 1.15 mM). However, unlike AST, potassium levels were also elevated in the 24-h-CI livers (6.62 ± 1.40) compared with the fresh livers (5.13 ± 0.30; p < 0.0003 between all groups at t = 0 min).

At the end of perfusion, we analyzed the energy status of the liver tissue based on the ratio of adenosine triphosphate (ATP), adenosine biphosphate (ADP), and adenosine monophosphate (AMP) (Fig. S2). This is known as the adenylate energy charge which has been correlated to transplant survival in the used animal model^20^ as well as to graft function after clinical liver transplantation^42,43^. At the end of perfusion, the energy charge of fresh (0.28 ± 0.05) and 24-h-CI livers (0.27 ± 0.07) was significantly higher than the energy charge of 72-h-CI livers (0.14 ± 0.04; p < 0.0170 and p < 0.0142, respectively), which agrees with our previous findings^20^.

### Morphologic analysis of cold liver ischemic livers after perfusion

Additionally, we performed histological analysis of the liver tissue at the end of perfusion (Fig. S3). Fresh controls showed normal lobular architecture without signs of injury. Liver lobules of the 24-h-CI livers showed well preserved architecture with mostly patent sinusoids and conspicuous LSECs. Mild congestion of liver sinusoids and focal disruption of endothelial lining with detached cells in central veins and cellular debris in the lumen was seen. Hepatocytes showed mild reactive changes in the form of scattered binucleated hepatocytes and hydropic degeneration with scarce hepatocyte dropout. The 72-h-CI livers showed preserved lobular architecture, although congestion of the sinusoids was evident. Focal disruption of endothelial lining was present and detached cells, eosinophilic granular and cellular debris were seen the lumen of both portal and central veins. LSECs appeared loosely attached and displaced by perisinusoidal subendothelial edema in the space of Disse. Reactive hepatocytes changes and parenchymal drop out with patchy centrilobular hydropic degeneration were more present as compared to 24-h-CI livers.

### Correlations between cell release and biochemical parameters of liver function and injury

**Figure 5:**
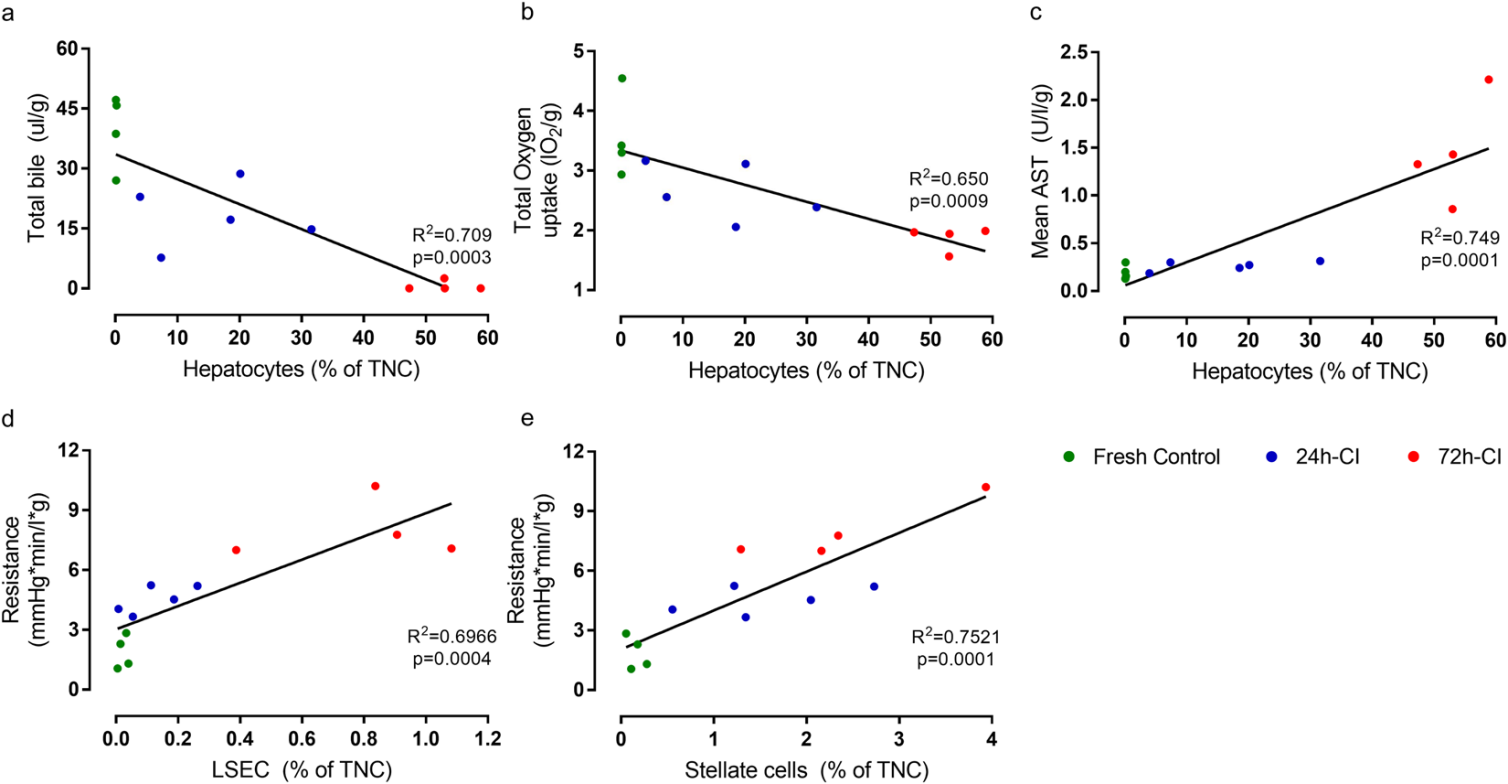
Correlations between structural liver cell release and corresponding viability parameters. Green: fresh livers (n = 4). Blue: 24-h-cold ischemic (CI) livers (n = 5). Red: 72-h-CI livers (n = 4). Black lines: fits of linear regression with 1, 11 degrees of freedom (DFn, DFd). Cell types are expressed as a percentage of the total number nucleated cells (TNCs) released into the perfusate. (**a**) Total bile production vs. percentage of *presumed* hepatocytes released into the perfusate that recirculated during 3 h of subnormothermic machine perfusion (SNMP). (**b**) Total oxygen uptake (area under the curve) vs. percentage of *presumed* hepatocytes released into the perfusate during 3 h of SNMP. (**c**) Mean aspartate aminotransferase (AST) concentration in the hepatic vein vs. the percentage of *presumed* hepatocytes released into the perfusate during 3 h of SNMP. (**d**) Vascular resistance at the start of perfusion vs. the percentage of *presumed* liver sinusoidal endothelial cell (LSECs) released in the perfusate that flushed once through the liver at the start of perfusion. (**e**) Vascular resistance at the start of perfusion vs. the percentage of *presumed* stellate cell released at the start of perfusion.

Because ischemia leads to hepatocyte injury and hepatocytes are solely responsible for bile excretion, we hypothesized that bile production could be related to hepatocyte release. We found a significant negative correlation between total bile production and the percentage of hepatocytes^p^ in the perfusate (p = 0.0003, R^2^ = 0.709; linear regression throughout the text) (Fig. 5a). Because 80% of the liver cells are hepatocytes, they represent the bulk of oxygen uptake measured for the whole liver. Therefore, we also tested the relationship between total oxygen uptake (AUC) and hepatocyte release during perfusion and found a significant negative correlation (p = 0.0009, R^2^ = 0.650) between them (Fig. 5b). Similarly, we found a significant positive correlation between AST levels and hepatocyte^p^ release (p = 0.0001, R^2^ = 0.749) (Fig. 5c).

Whereas hepatocyte^p^ release during perfusion was most strongly related to the degree of ischemic injury during HP, LSEC^p^ and stellate^p^ cell releases were more indicative of cold ischemic injury in the perfusate fractions collected at the very start of perfusion (Fig. 4). LSEC and stellate cells play a crucial role in liver microcirculation^38,44^, which is most reflected by vascular resistance during perfusion. Therefore, we expected a positive correlation between LSEC and stellate cell release with vascular resistance at the start of perfusion. Indeed, the release of both LSEC^p^ and stellate^p^ cells in the perfusate fractions collected at the start of perfusion were strongly and significantly correlated to vascular resistance at the start of perfusion (p = 0.0004, R^2^ = 0.6966 and p = 0.0001, R^2^ = 0.7521, respectively) (Fig. 5d,e, respectively).

## Discussion

The global donor organ shortage indirectly claims hundreds of thousands of lives each year in the US alone^3^. There is a large heterogeneous pool of donor livers of uncertain quality, which, if tapped, could dramatically reduce the gap between supply and demand^4^. However, the use of those organs incurs an increased risk of negative transplant outcomes. Hence the selection of low-risk donor livers to avoid transplant failures and more importantly, the lack of technology to accurately assess graft viability, have precluded the use of a substantial pool of potentially viable donor livers every year. We proposed that liver-specific cell release into the HP solution and into the perfusate during machine perfusion could serve as novel biomarkers to assess organ viability and identify injury mechanisms. Our results confirmed that tissue injury during preservation leads to the release of structural liver cells (Fig. 2) and alterations in the release of liver-resident immune cells (Fig. 3). Although endothelial cell detachment has been studied as a marker of vascular injury^45^, we show here for the first time, that parenchymal cells are also released from organs under non-proliferative pathological conditions. This may have widespread consequences for many pathological conditions beyond tissue injury during organ preservation.

The first concern was that there were not enough cells in the perfusate. Instead we found that millions of cells were released during perfusion (Fig. 1). In this study, we processed the complete perfusate volume for imaging flowcytometry analysis. Based on the result that 400 ml of perfusate contained approximately 4 M cells (Fig. 1), a 2 ml perfusate sample would provide 20,000 cells, which is sufficient for imaging flowcytometry analysis. Moreover, the perfusate-to-tissue volume ratios are approximately 50 times larger in human liver perfusion compared with rat liver perfusion (1:1 vs. 1:50 g_tissue_/ml perfusate, respectively), which suggests that sample volumes as little as 50 µl may potentially be used for analysis. Microfluidic chip technology could likely provide real-time measurement in just a droplet of perfusate.

We expressed the cell release data as a percentage of the TNCs instead of absolute numbers of specific cell types (although these data are also relevant and have been provided in Table S2). Such an approach has multiple important advantages. First, it reduces the standard error of liver-to-liver variances in overall cell release. Second, the percentage of TNCs provides a metric that is independent of liver size and perfusate volume. Such metrics facilitate the translation from animal models to human studies because variation in organ size does not change the percentage of liver specific cells whereas it most likely will change the absolute numbers of released cells. The size indifference is additionally important for translational efforts because human livers vary up to 300% in liver weight between donors^46^, and different flush and perfusate volumes are used in clinical protocols.

Although the released cells during perfusion also included remnant peripheral white blood cells, a substantial proportion (~20–75% dependent on liver viability; Figs. 2a and 3a) of the released cells were liver specific and included both structural and resident immune cells. The identity of these liver specific cell population was *presumed* based on surface marker expression as indicated by superscript *p* throughout the manuscript. For the structural cells (Fig. 1), we focused on hepatocytes, LSECs, and stellate cells and were guided by our hypothesis that they were most likely to be released through their pericapillary anatomical localization. The three cell types were nearly absent in the perfusate of fresh livers and all three significantly increased as a function of cold ischemic injury. Most importantly, in our *ex vivo* model, the release of hepatocytes^p^ and stellate^p^ cells during perfusion discriminated fresh, mild and severe ischemic livers with strong statistical significance (Fig. 1b).

As a potential mechanism of structural cell release, it is know that ischemia-reperfusion is associated with increased tissue levels of proteolytic enzymes^16,47^. We hypothesize that these enzymes may injure the extracellular matrix, causing loss of local tissue integrity and subsequent cell detachment. In addition to these biochemical alterations, we hypothesize that mechanical shear stress - exerted on the structural cells during the flush and perfusion - plays an important role in cell detachment. Together this could explain the focal disruption of tissue architecture and detached cells that were seen in the vascular lumen on histology (Fig. S3) and the structural cells that were detected in the perfusate, although further studies are required to confirm these hypotheses.

Of all the liver-specific structural cell releases that were measured during perfusion, hepatocyte^p^ release was the most sensitive marker of ischemic injury during hypothermic preservation. This may be due to the large number of hepatocytes in livers. Based on previous literature, rat livers contain about 1.4 × 10^9^ hepatocytes^48^. This is over one thousand times the number of hepatocytes^p^ that were released after severe ischemia, and over one million times the number of hepatocytes^p^ that were released from fresh livers (Table S2). This indicates that hepatocyte^p^ release can be detected with high sensitivity. As a corollary question, we analyzed if hepatocyte^p^ release was related to the alterations in hepatocyte function and injury as result of cold ischemia. We found highly significant correlations between hepatocyte release and bile production, AST release, and oxygen uptake during perfusion. This demonstrates that specific markers of hepatocyte and function may be reflected by hepatocyte^p^ release.

Although machine perfusion is an emerging preservation technology, most transplanted livers are not machine perfused because HP is still the current clinical standard. Cells may also be released in the HP storage solution and could potentially be analyzed without the need of machine perfusion. In this regard, we found that hepatocytes^p^, LSECs^p^ and Kupffer^p^ cells (Fig. 3) were released at the very start of perfusion and that this markedly changed as result of cold ischemia. This very early release of cells during perfusion may suggest that these cells were already released in the HP solution, however, they also could have been released due to perfusion with the oxygenated perfusate. Therefore, it remains to be confirmed if cells are released in the HP storage solution and if they could be used as biomarkers without the need of machine perfusion.

In addition to its diagnostic potential, the measurement of cell release from livers may also provide insights into the specific injury mechanisms that occur during preservation. This is supported by our results that showed the release of LSECs^p^, stellate^p^, and Kupffer^p^ cells during perfusion. LSECs are known to be especially sensitive to transplant-induced ischemia-reperfusion injury, although the mechanism of injury during HP is not fully understood^49–51^. The changes in LSEC^p^ release during perfusion (Figs. 2a and 4b) provided new insights into the mechanism of endothelial injury, which could be used to improve machine perfusion protocols. LSEC injury is known to reduce liver viability and machine perfusion is thought to protect against and alleviate the effects of endothelial injury. This is consistent with our results that showed very low LSEC^p^ release from fresh livers throughout perfusion. However, increasing LSEC^p^ release was seen during machine perfusion of ischemic livers. This suggests that machine perfusion could be damaging to the endothelium if the LSECs are already injured by ischemia. Such novel insights could help improve machine perfusion protocols of ischemic livers, and additional protective strategies may be employed to prevent LSEC injury.

Knowledge about the role of stellate cells in organ transplantation is much more limited^52,53^; however, their importance is increasingly acknowledged in disease entities such as liver fibrosis^54^ and steatoses^55^, which are important exclusion criteria for organ donation. Here, we showed that stellate^p^ cells were significantly injured during graft preservation (Figs. 2a and 4c) and their release into the perfusate is correlated to increased vascular resistance (Fig. 5e). Future studies should investigate the effects of stellate cell injury during preservation.

We found that the percentages of liver-resident immune cells that were released in the perfusate showed a declining trend with increasing durations of ischemia (Fig. 3), as opposed to the above discussed increasing numbers of structural cells (Fig. 2). This effect was strongest for the Kupffer^p^ cell percentages which were highest in the perfusates of fresh livers and significantly decreased as a function of increasing CI time. This may be explained by Kupffer cell activation and infiltration during cold ischemia. Kupffer cells have an important mediating role in ischemia-reperfusion (I/R) injury, which is a major cause of primary non-function and early allograft dysfunction^56^. While machine perfusion has shown to reduce I/R injury during transplantation via multiple pathways, one of the proposed mechanisms is that cytokines are diluted in the perfusate. This results in reduced Kupffer cell activation and infiltration^57^, as was seen in histology samples of hypothermic preserved and machine perfused grafts^58^. However, in the present study, we showed that Kupffer^p^ cells were washed-out during machine perfusion (Fig. 4d and Table S2), which may be another reason for reduced I/R injury after machine perfusion.

As this is a proof of principle study, limitations with respect to the used *ex vivo* animal model should be considered regarding the translation toward clinical application of cell release-based viability assessment. First, whereas hypothermic and normothermic machine perfusion recently entered the clinic, SNMP remains in pre-clinical phase. Although the assessment of injury and function during SNMP has strong theoretical background^21,40,58,59^, the validity of this model to infer clinical post-transplant outcomes is limited. Second, the 24-h and 72-h durations of CI were chosen to represent transplantable and non-transplantable livers based on our previous results after transplantation using the exact same animal model^20,21^. However, the livers were not transplanted in the present study. Although the outcomes after transplantation would have very likely been the same, significant correlations with post-transplant graft survival and function cannot be inferred from the results. A final consideration is that rat livers are more resilient to ischemia than human livers. Despite these limitations the main findings of this study encourage future clinical studies to assess cell release as a predictor of post-transplant clinical outcomes.

Future studies may incorporate composite cell indices using a combination of liver cell types to account for differences in susceptibilities and different injury mechanisms. For example, sinusoidal endothelial cells have been shown to be more susceptible to cold ischemia, whereas warm ischemia seems more injurious to Kupfer cells^60^. A composite index may therefore more accurately capture the multimodal injury mechanisms in human liver transplantation in future clinical studies. Moreover, future studies may also provide a deeper understanding of injury and protective mechanisms during preservation. Using flow cytometry fluorescence activated cell sorting (FACS), each specific released cell type can be isolated for further biomolecular analysis. Almost no structural liver cells were released from healthy tissues (Fig. 2); therefore, the isolated cells may be used to specifically identify injury-derived expression signatures. Also, these expression signatures will represent the entire graft and therefore, are not prone to spatial differences. Together, these are important advantages over existing methods, such as tissue biopsies, exosome release, or cell-free DNA/RNA analysis, and may therefore yield important additional information during future investigation.

Finally, other solid transplant organs may also release cells as result of ischemia during preservation. This may be leveraged in similar ways to obtain markers of graft injury and gain deeper understanding of the molecular injury mechanisms during handling and preservation of donor organs. In combination with rapidly advancing rare-cell sorting techniques – initially developed for detecting circulating tumor cells in peripheral blood^61^ – these non-proliferative cell release-based biomarkers might also be used to detect organ injury *in vivo* after transplantation. The feasibility of detecting and isolating circulating hepatic epithelial cells was recently demonstrated and shown to be indicators for the transition of chronic liver disease into hepatocellular carcinoma^62^. These findings together with our discovery that parenchymal cells are released and detectable after injury under non-proliferative conditions can potentially have important implications. Structural injury to tissues and organs is a widespread cause of medical conditions. Therefore, this concept of parenchymal cell release could facilitate the identification of novel biomarkers for applications beyond organ transplantation.

## Materials and Methods

### Experimental design

The donor livers were procured and subjected to HP for 0 (fresh) (n = 4), 24 h (n = 5), and 72 h (n = 4), after which the livers underwent 3 h of SNMP. Allocation of the livers was arbitrarily alternated between the experimental groups. The perfusate was collected and analyzed using multi-channel imaging flow cytometry to detect hepatocytes^p^, sinusoidal endothelial^p^, stellate^p^, Kupffer^p^, pit^p^ (liver-associated natural killer), and dendritic^p^ cells (superscript p denotes cells are *presumed* based on surface antigen expression). The first 100 ml and last 100 ml of perfusate that flushed through the livers were collected separately from the 400 ml recirculating perfusate to study the changes in cell-release profiles (Fig. 1).

### Animal care

Livers from healthy male Lewis rats (Charles River Laboratories, Wilmington, MA, USA) were used for all experiments to ensure comparable baseline characteristics between the experimental groups (Table S3). The animals were housed in a temperature- and humidity-controlled room equipped with a natural light/dark cycle, socially housed (according to weight standards) with conventional bedding, and were provided with free access to standard food and water – in accordance with the National Research Council guidelines. The health and welfare of the animals was maintained by Massachusetts General Hospital Center of Comparative Medicine (CCM), and the experimental protocols were approved by the Institutional Animal Care and Use Committee (IACUC) of Massachusetts General Hospital (Boston, MA, USA).

### Liver procurement

The rats (200–250 g) were anesthetized with isoflurane in a dedicated animal surgery laboratory room. The liver was exposed through a transverse abdominal incision and freed from its connecting ligaments. The bile duct (BD) was dissected and cannulated with a 28-gauge catheter using a surgical microscope. Next, the portal vein (PV) was cannulated past the gastroduodenal and splenic branches with a 16-gauge catheter. The cannulas of the PV and BD were secured using 3.0 silk sutures. After the hepatic artery was identified and tied off, the infrahepatic inferior vena cava (IHVC) was transected and the liver was flushed with 60 ml of ice-cold saline. While still under anesthesia, the rats were euthanized by exsanguination from the IHVC. Finally, the suprahepatic inferior vena cava and hepatoduodenal ligament were transected, and the liver was freed from the remaining ligaments and removed from the abdomen.

### Hypothermic preservation

Directly after procurement, the liver for the 24-h-CI and 72-h-CI groups were flushed with 30 ml of ice-cold University of Wisconsin (UW) solution (Bridge to Life, Columbia, SC, USA). Next, the livers were submerged in UW solution and stored at 4 °C in a sealed bag.

### Subnormothermic machine perfusion

The machine perfusion system consists of non-pulsatile circulation providing portal perfusion through the liver. The perfusate is pumped from a 500-ml reservoir bottle by a flow-rate controlled roller pump (Cole Palmer, Vernon Hills, IL, USA) through an oxygenator (Radnoti, Monrovia, CA, USA), bubble trap (Radnoti), pressure sensor (Living Systems Instrumentation, Albans City, VT, USA), sampling port (Cole Palmer), and finally, an organ chamber that holds the liver during perfusion. The components are connected in consecutive order with size-16 silicone tubing (Cole Palmer). The oxygenator is supplied with a gas mixture of 95% O_2_ and 5% CO_2_, and the perfusion temperature is maintained at an ambient temperature of 21 °C (±1 °C).

Prior to perfusion, the system was primed with 500 ml of perfusate. The perfusate consisted of powdered Williams Medium E (Sigma-Aldrich, St Louis, MO, USA) supplemented with sodium bicarbonate (2.2 g/l; Sigma-Aldrich), dexamethasone (24 mg/l; Sigma-Aldrich), insulin (5 U/l; MGH Pharmacy), heparin (2000 U/l; MGH Pharmacy), and bovine serum albumin (10 mg/ml; Sigma-Aldrich). The bubble trap was filled to 25% with perfusate and therefore also served as a compliance chamber to minimize pressure pulses created by the roller pumps. The system was run freely for ~15 min to oxygenate the perfusate and adjust the pH to 7.3–7.4 with sodium bicarbonate, if necessary. During this period, pressure sensor was calibrated using a dummy 16-gage catheter (the same cannula that was used to cannulate the PV) for flow rates from 0 to 30 ml/min.

The perfusion was initiated by attaching the PV cannula to the perfusion system. The outflow of the BD canula was placed in a 1.5-ml Eppendorf tube to collect the produced bile. During the first 30 min of perfusion, the pressure over the liver was gradually increased to 5 mmHg and regulated throughout perfusion by manually adjusting the flow rate of the pump, up to a flowrate of maximum 25 ml/min. The liver drained freely into the organ chamber and the first 100-ml return of the organ chamber was collected in a 100-ml graded cylinder before closing the circuit. The remaining perfusate was recirculated by directing the return of the organ chamber to the perfusate reservoir that closed the perfusion circuit. After 3 h of perfusion, the perfusate reservoir was replaced with a fresh perfusate bottle. This created a small bubble in the system that was followed until it was caught in the bubble trap. At this moment, the return of the organ chamber was diverted into a fresh bottle, and the last 100 ml of fresh perfusate was collected separately.

### Imaging flowcytometry

The hepatocytes^p^, LSECs^p^, stellate^p^, Kupffer^p^, pit^p^, and dendritic^p^ cells in the perfusates were studied using the ImageStreamX Mark II imaging flow cytometer (Amnis Corporation, Seattle, WA, USA) equipped with 405-, 488-, and 642-nm lasers, a 40× objective, and six imaging channels. To identify the six different cell types, we used three flowcytometry panels for the detection of two cell types each: panel 1 for the detection of hepatocytes and LSECs; panel 2 for the detection of stellate cells and Kupffer cells; panel 3 for the detection of pit cells and dendritic cells.

Within 1 h after the end of SNMP, half the volumes of the three perfusates were spun down at 200 *g* and resuspended in a total volume of 1.5 ml. For each of the three perfusate fractions, three 200-µl aliquots of the 1.5-ml cell suspension were stained for the different flowcytometry panels as follows.

All aliquots were stained with DRAQ (1:200; Biolegend, Deadham, MA, USA; Cat# 424101) and PECy7-conjugated CD45 antibody (1:50; Biolegend, Cat# 202214). The aliquot for panel 1 was additionally stained for the detection of hepatocytes^p^ and LSECs^p^ with Alexa 405-conjugated SE1 (1:100; Novus Biologicals, Centennial, CO, USA; Cat# NB110-68095AF405), FITC-conjugated ASGR1 (1:50; Miltenyi Biotec, Cambridge, MA, USA; Cat# 130-109-490), and PE-conjugated OX62 (1:100; Thermo Fisher, Waltham, MA, USA; Cat# 12-1030-82) antibodies.

The aliquot for panel 2 was additionally stained for the detection of stellate^p^ and Kupffer^p^ cells with Alexa 405-conjugated CD105 (1:87; Novus Biologicals; Cat# NB500-452AF405), FITC-conjugated SE1 (1:100; Novus Biologicals; Cat# NB110-68095F), and Cy3-conjugated CD14 (2:100; Bioss, Woburn, MA, USA; Cay# bs-1192R-Cy3) antibodies.

The aliquot for panel 3 was additionally stained for the detection of pit^p^ cells and dendritic^p^ cells with Alexa 405-conjugated NKRP1A (1:80; Novus Biologicals; Cat# NB100-65297AF405), FITC-conjugated CD3 (1:50; Invitrogen, Carlsbad, CA, USA; Cat# 11-0030-82), and PE-conjugated OX62 (1:100; Thermo Fisher; Cat# 12-1030-82) antibodies.

All stains were incubated at room temperature for 30 min except DRAQ, which was incubated for 10 min. Directly after incubation, each of the total of nine samples per perfusion was run separately in the imaging flow cytometer with the laser powers set at 100, 100, and 150 mW for the 405-, 488-, and 642-nm lasers, respectively.

### Imaging flow cytometry data processing

Flow cytometry data was processed using the IDEAS 6.2 (Amnis Corporation) software package. Out of all the recorded events, we selected the normal-shaped and -sized events (NSS) using area and aspect ratio of the brightfield channel. Hence only whole cells are included in subsequent analysis and smaller events such as cell debris or extracellular vesicles are excluded. Out of these NSS events, we selected the DRAQ-positive events to obtain the TNCs. To select the cells of interest more accurately, we first selected all TNCs that were positive for at least one of the remaining channels and used this potential cell-of-interest (PCI) population for further analysis.

For panel 1, we selected the hepatocytes^p^ as ASGR1^+^/SE1^−^ cells from the PCI population and confirmed that those cells were CD45^−^ and OX62^−^. LSECs were selected from the same PCI population as ASGR1^−^/SE1^+^ cells and confirmed that they were also CD45^−^ and OX62^−^.

In panel 2, we selected the stellate^p^ cells in two steps: first, we gated CD105^+^/CD14^+^ cells from the PCI population; then, we selected the stellate cells as CD45^−^/SE1^−^ cells from this subpopulation. To select the Kupffer^p^ cells, we gated the CD14^+^/CD105^−^ population and confirmed that these cells were CD45^+^ and SE1^−^. Because CD14 expression is influenced by Kupffer cell activation^30,32^, we used an intensity threshold of 1e4 to define CD14^+^ cells, as illustrated in Fig. 2b, top.

In panel 3, we selected the pit^p^ cells as the NKRP1A^+^/CD3^−^ cells from the PCI population and confirmed that those cells were CD45^+^ and OX62^−^. We selected the dendritic^p^ cells from the PCI in three steps. In the first two steps we selected the NKRP1A^−^/CD3^−^ cells from the CD45^+^/OX62^+^ cells in the PCI population. Because dendritic^p^ cells are rare, we then manually selected the true CD45^+^/OX62^+^/NKRP1A^−^/CD3^−^ cells from this subpopulation.

Relative numbers of the specific cell types in the perfusate were calculated by dividing the number of each cell type by the number of TNCs in each panel. Absolute numbers of the specific cell types in the perfusate were calculated as follows: the count of each cell type was divided by the volume processed by the flow cytometer to obtain the specific cell concentrations in the 200-µl flowcytometry aliquot. This concentration was multiplied by the centrifugation dilution factor and by the total volume of the perfusate (i.e., either 100 or 400 ml) to obtain the total number of specific cell types in the perfusate.

### Optimization and validation of imaging flowcytometry analysis

For optimization of the imaging flow cytometry analysis, we verified specificity of the antibodies and optimized antibody concentration as well as incubation time using purified populations of rat liver cells obtained from a two-step EDTA collagenase procedure from the Cell Resource Core (Massachusetts General Hospital, Boston, MA, USA) as per established protocols^63,64^. In this capacity, positive and negative controls were derived from purified cell fractions of either hepatocytes, LSECs, stellate cells, Kupffer cells, or a fraction containing all non-parenchymal cells for identification of Pit and dendritic cells. Further, we also assessed the influence of other variables on antibody performance including different antibody clones, fluorophores, and vendors.

### Perfusion data acquisition

The liver was weighed directly after procurement, HP, and SNMP. Real-time perfusate measurements were performed every 30 min; pH and pO_2_ were measured in the PV and IHVC, and the potassium concentration was only measured in the IHCV using an i-STAT blood analyzer (Abbot Laboratories, Chicago, IL, USA). Additional perfusate samples were taken from the IHVC, immediately frozen on dry ice, and stored at −80 °C for post-hoc analysis of AST activity using a colorimetric kit (ThermoFisher Scientific, Pittsburgh, PA) according to the manufacturers’ instructions. Cumulative bile production was measured by weighing the bile-containing Eppendorf tube on a microscale.

For histological analysis, tissue biopsies were taken directly after perfusion, fixed in buffered 5% (v/v) formaldehyde for 24 hours, and stored in 70% (v/v) ethanol. Tissue processing and staining for hematoxylin and eosin (H&E) was performed at the Massachusetts General Hospital Histology Molecular Pathology Core Facility, Boston, MA, USA. Histology slides were assessed by an experienced liver pathologist (E.O.A.H). For analysis of adenylate energy charge, additional (approx. 1 gr) tissue biopsies were flash frozen in liquid nitrogen and stored at −80 °C. Measurement of the adenylate energy charge by liquid chromatography-mass spectrometry (LC-MS) was performed at the Shriners Hospitals-Boston Mass Spectrometry Core Facility, Boston, MA.

### Perfusion data processing

To calculate vascular resistance in the PV, the perfusion pressure was divided by the corresponding flow rate that was multiplied by the weight of the liver after procurement.

Oxygen concentrations in the outflow (IHVC) and the inflow (PV) were derived from Henry’s law, CdO_2_ = aO_2_ × PO_2_, where CdO_2_ is the concentration of dissolved oxygen, aO_2_ is the solubility coefficient for oxygen (0.00314 ml O_2_/mmHg O_2_/dl blood), and PO_2_ is the partial oxygen pressure that was measured in the PV and IHVC during SNMP. To calculate the oxygen uptake rate (OUR), oxygen concentration of the outflow was subtracted from that of the inflow; this difference was multiplied by the flow rate and finally divided by the weight of the liver after procurement.

### Statistical analysis

Repeated measures two-way ANOVAs were used for the comparison of the time-course perfusion and cell-release data, followed by Tukey’s post-hoc test to examine statistical differences between the experimental groups and to correct for multiple comparisons. Total oxygen uptake was calculated by integration of the oxygen uptake rate to calculate the area under the curve (AUC). Total oxygen uptake was compared with the Kruskal-Wallis test, followed by Dunn’s post-hoc test. Linear regressions were used to correlate the viability parameters to cell release during machine perfusion. All statistical analyses were performed with Prism 7.03 (GraphPad Software Inc., La Jolla, CA) with a (two-sided) significance level of 0.05. Adequacy of the statistical analysis was endorsed by an experienced Biostatistician (A.M.).

## Supplementary information

Supplementary materials.

## Data Availability

The authors declare that the data supporting the findings of this study are available within the paper and its supplementary information files. Any additional data, if needed, will be provided upon request.
