# Supplementary material for: Cell release during perfusion reflects cold ischemic injury in rat livers
Source: Sci Rep. 2020 Jan 24;10:1102. doi: 10.1038/s41598-020-57589-4 (PMC6981218; doi:10.1038/s41598-020-57589-4)
Supplement: Supplementary file 1 — Supplementary materials. [file 41598_2020_57589_MOESM1_ESM.pdf]

Cell release during perfusion reflects cold ischemic injury in rat livers

Reinier J. de Vries[1,2,3], Casie A. Pendexter[1,2], Stephanie E.J. Cronin[1,2], Beatriz Marques[1], Ehab O.A. Hafiz[1,2,4], Alona Muzikansky[5,6], Thomas M. van Gulik[3], James F. Markmann[7], Shannon L. Stott[1,6], Heidi Yeh[7], Mehmet Toner[1,2], Korkut Uygun[1,2], Shannon N. Tessier[1,2,*]

[1]*Center for Engineering in Medicine, Harvard Medical School and Massachusetts General Hospital, Boston MA, USA*

[2]*Shriners Hospitals for Children - Boston, Boston MA, USA*

[3]*Department of Surgery, Amsterdam University Medical Centers – location AMC, University of Amsterdam, Amsterdam, the Netherlands*

[4]*Department of Electron Microscopy Research, Theodor Bilharz Research Institute, Giza, Egypt.*

[5]*Biostatistics Center, Massachusetts General Hospital, Boston, MA, USA*

[6]*Cancer Center, Massachusetts General Hospital and Harvard Medical School, Boston MA, USA*

[7]*Division of Transplant Surgery, Massachusetts General Hospital, Boston MA, USA*

[*]Corresponding author:

Shannon N. Tessier

Instructor, Center for Engineering in Medicine

Massachusetts General Hospital and Harvard Medical School

Tel: 617-952-9192

Email: sntessier@mgh.harvard.edu

Supplementary Figures and Tables

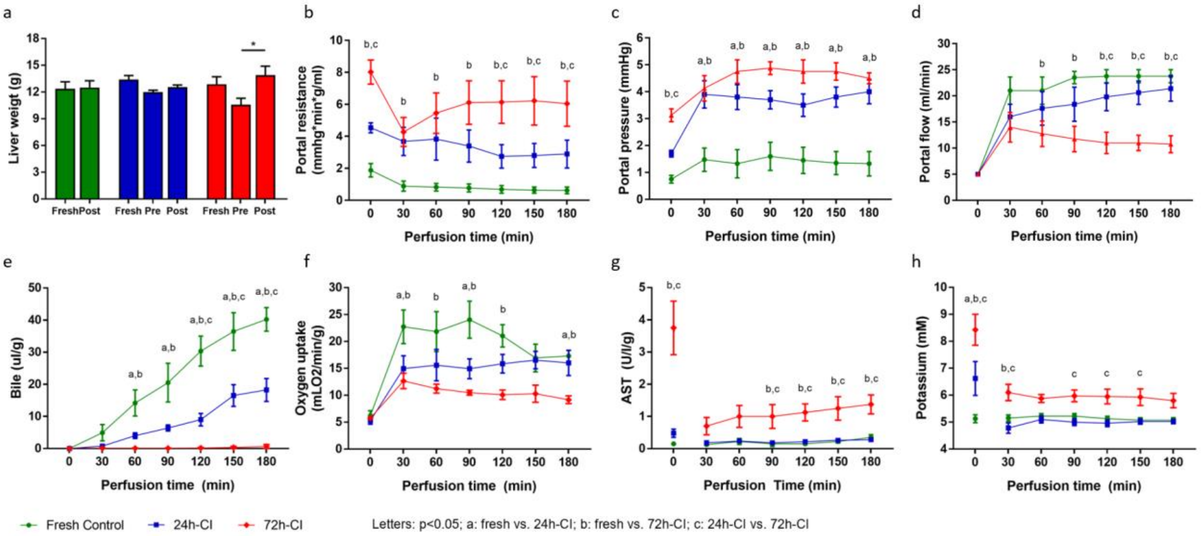

Figure S1: Parameters of liver function and injury during subnormothermic machine perfusion. Green: fresh livers (n = 4). Blue: 24-h-cold ischemic (CI) livers (n = 5). Red: 72-h-CI livers (n = 4). **a.** Liver weight directly after procurement, before and after perfusion (fresh, pre, and post, respectively) **b.** Vascular resistance of the portal vein. **c. P**erfusion pressure in the portal vein. **d.** Portal flow. **e.** Cumulative bile production. **f.** Oxygen uptake calculated from the partial oxygen pressure in the portal and hepatic veins. **g.** Aspartate aminotransferase (AST) concentration in the hepatic vein. **h.** Potassium concentration in hepatic vein. Note that the initial drop in AST and potassium between t = 0 and 30 min is due to the nature of the experimental design whereby the first fraction of perfusate was removed for imaging flow cytometry. Letters and star denote statistical significance (repeated measures two-way ANOVA, followed by Tukey's post-hoc test; $p < 0.05$); a: fresh vs. 24-h-CI; b: fresh vs. 72-h-CI; c: 24-h-CI vs. 72-h-CI.

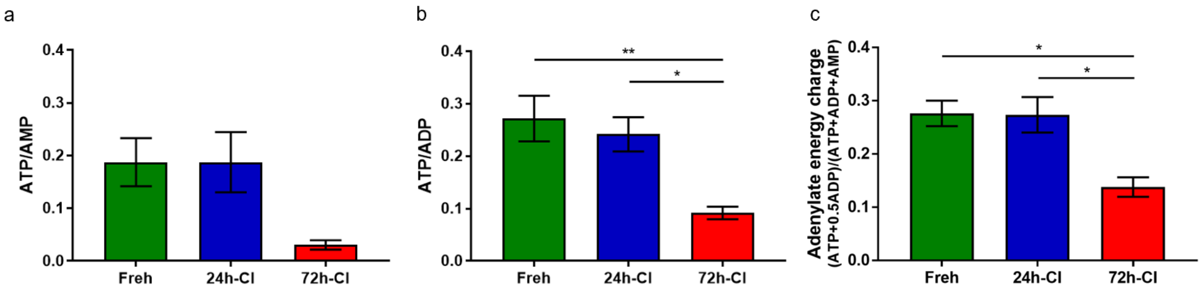

Figure S2: Energy status of liver tissue after cold ischemia and subsequent subnormothermic machine perfusion. Green: fresh livers (n = 4). Blue: 24-h-cold ischemic (CI) livers (n = 5). Red: 72-h-CI livers (n = 4). The energy status was measured in biopsies taken directly after sub normothermic machine perfusion. **a.** Ratios between adenosine triphosphate (ATP) and adenosine monophosphate (AMP). **b.** Ratios between ATP and adenosine biphosphate (ADP). **c.** Adenylate energy charged, defined by the ratio between (ATP+0.5*ADP) and (ATP+ADP+AMP). Stars denote statistical significance (repeated measures two-way ANOVA, followed by Tukey's post-hoc test): *0.01 < p < 0.05; **0.001 < p < 0.01. Error bars: SEM.

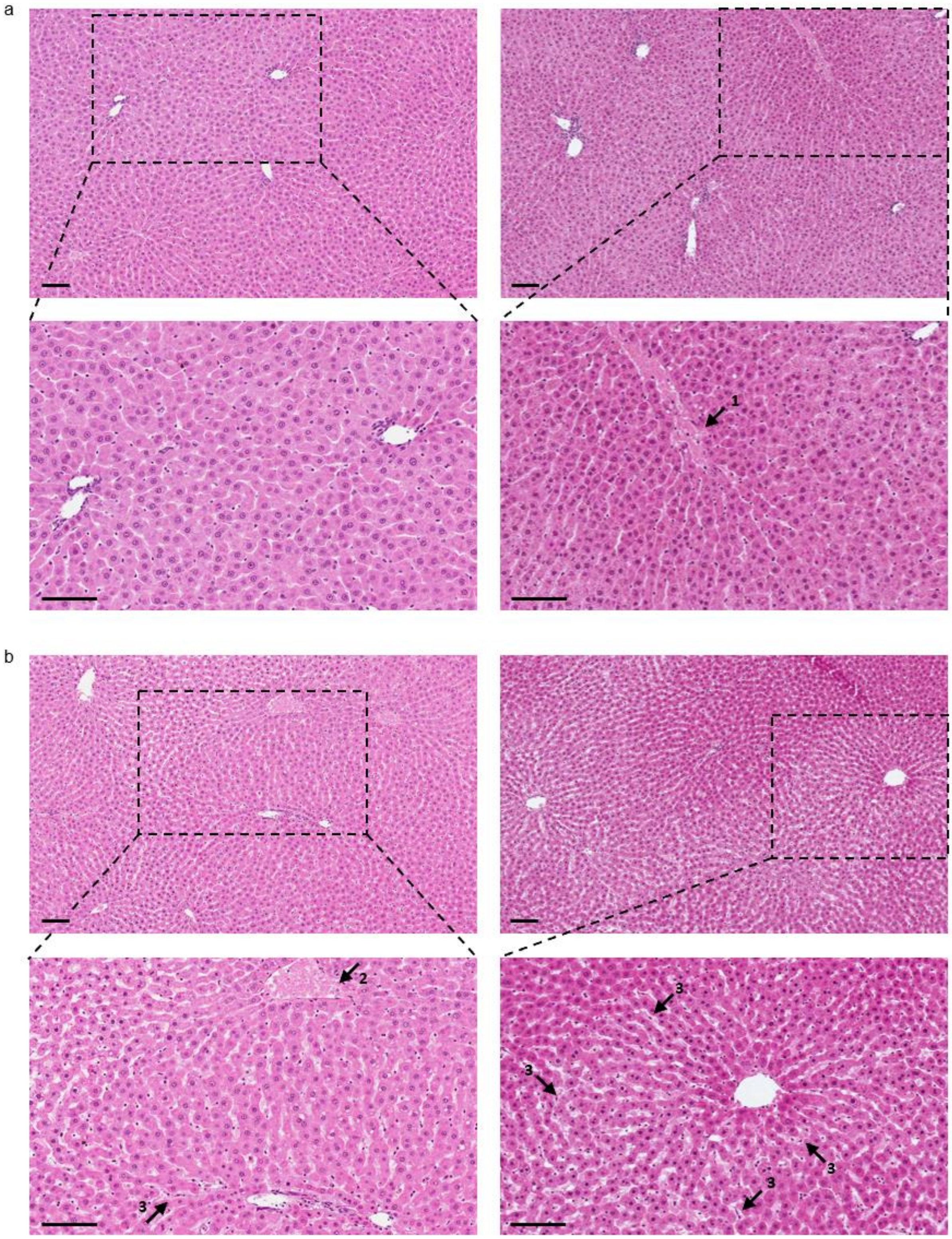

Figure S3: Morphological analysis after cold ischemia and subsequent subnormothermic machine perfusion. Light microscopy images of parenchymal liver biopsies stained for hematoxylin and eosin staining taken directly after perfusion. **a.** Representative images for 24-h-CI livers. Left: well preserved lobular architecture with mostly patent sinusoids and conspicuous LSEC. Right: Focal mild congestion

of liver sinusoids. Arrow 1: focal disruption of the endothelial lining with detached cells and cellular debris in the lumen. **b.** Representative images for 72-h-CI livers. Arrow 2: Focal disruption of endothelial lining with detached cells, eosinophilic granular and cellular debris in the central vein lumen. Arrows 3: LSECs appear very loosely attached and displaced by perisinusoidal subendothelial edema. Scale bars: 100 µm.

Table S1: Relative surface marker expression on all released nucleated cells

	Fresh	24h-CI	72h-CI
Surface Marker	*% (Mean ± SD)*	*% (Mean±SD)*	*% (Mean±SD)*
**CD45**	94.59 ± 5.92	51.20 ± 19.65	15.77 ± 6.27
**ASGR1**	0.48 ± 0.48	19.22 ± 11.98	57.45 ± 5.29
**SE1**	0.23 ± 0.23	11.16 ± 9.34	15.43 ± 10.60
**CD105**	4.36 ± 2.20	18.29 ± 8.73	45.10 ± 21.72
**CD14**	8.98 ± 2.31	22.48 ± 11.43	49.94 ± 22.48
**NKRP1A**	18.23 ± 2.33	17.99 ± 10.22	22.55 ± 8.14
**CD3**	45.75 ± 7.54	25.10 ± 10.39	13.57 ± 9.44
**OX62**	2.38 ± 0.29	4.21 ± 1.67	9.16 ± 4.98

Note: Percentage is relative to the total number of nucleated cells that are released into the perfusate of fresh, 24-h-cold ischemic (CI), and 72-h-CI livers. Cells can express more than one marker.

Table S2: Absolute number of liver-specific cells released into the perfusate

	Fresh	24h-CI	72h-CI
Cell type	*(Mean ± SD)*	*(Mean ± SD)*	*(Mean ± SD)*
**Hepatocytes**	$4.47\times10^3 \pm 1.95\times10^3$	$6.49\times10^5 \pm 6.76\times10^5$	$1.18\times10^6 \pm 5.44\times10^5$
**LSECs**	$3.74\times10^2 \pm 2.72\times10^2$	$2.66\times10^5 \pm 2.09\times10^5$	$2.56\times10^5 \pm 2.65\times10^5$
**Stellate Cells**	$4.49\times10^3 \pm 5.43\times10^3$	$3.37\times10^4 \pm 2.72\times10^4$	$5.55\times10^4 \pm 3.54\times10^4$
**Kupffer Cells**	$1.55\times10^5 \pm 4.47\times10^4$	$2.05\times10^4 \pm 8.64\times10^3$	$6.49\times10^3 \pm 4.67\times10^3$
**Pit Cells**	$5.72\times10^5 \pm 1.14\times10^5$	$4.95\times10^5 \pm 2.01\times10^5$	$3.21\times10^5 \pm 2.68\times10^5$
**Dendritic Cells**	$4.67\times10^3 \pm 1.58\times10^3$	$2.32\times10^3 \pm 3.58\times10^2$	$1.67\times10^3 \pm 8.22\times10^2$

Note: CI = cold ischemia.

Table S3: Animal characteristics of the experimental groups

Characteristic	**Fresh** *(mean ± SD)*	**24h-CI** *(mean ± SD)*	**72h-CI** *(mean ± SD)*
**Group size (n)**	4	5	4
**Species, Strain**	Rattus, Lewis	Rattus, Lewis	Rattus, Lewis
**Sex (% male)**	100%	100%	100%
**Age (weeks)**	14.4 ± 1.2	13.9 ± 1.2	14.3 ± 1.1
**Liver weight (grams)**	12.4 ± 1.6	13.4 ± 1.1	12.9 ± 1.7
